# A Multi-Task Convolutional Neural Network for Semantic Segmentation and Event Detection in Laparoscopic Surgery

**DOI:** 10.3390/jpm13030413

**Published:** 2023-02-25

**Authors:** Giorgia Marullo, Leonardo Tanzi, Luca Ulrich, Francesco Porpiglia, Enrico Vezzetti

**Affiliations:** 1 Department of Management, Production, and Design Engineering, Polytechnic University of Turin, 10129 Turin, Italy; 2 Division of Urology, Department of Oncology, School of Medicine, University of Turin, 10124 Turin, Italy

**Keywords:** multi-task convolutional neural network, CNN, semantic segmentation, bleeding detection, laparoscopic surgery

## Abstract

The current study presents a multi-task end-to-end deep learning model for real-time blood accumulation detection and tools semantic segmentation from a laparoscopic surgery video. Intraoperative bleeding is one of the most problematic aspects of laparoscopic surgery. It is challenging to control and limits the visibility of the surgical site. Consequently, prompt treatment is required to avoid undesirable outcomes. This system exploits a shared backbone based on the encoder of the U-Net architecture and two separate branches to classify the blood accumulation event and output the segmentation map, respectively. Our main contribution is an efficient multi-task approach that achieved satisfactory results during the test on surgical videos, although trained with only RGB images and no other additional information. The proposed multi-tasking convolutional neural network did not employ any pre- or postprocessing step. It achieved a Dice Score equal to 81.89% for the semantic segmentation task and an accuracy of 90.63% for the event detection task. The results demonstrated that the concurrent tasks were properly combined since the common backbone extracted features proved beneficial for tool segmentation and event detection. Indeed, active bleeding usually happens when one of the instruments closes or interacts with anatomical tissues, and it decreases when the aspirator begins to remove the accumulated blood. Even if different aspects of the presented methodology could be improved, this work represents a preliminary attempt toward an end-to-end multi-task deep learning model for real-time video understanding.

## 1. Introduction

Laparoscopy, widely known as minimally invasive surgery, is a surgical procedure that allows a surgeon to see within the abdomen and pelvis, without creating significant cuts [1], by having trocars with attached instruments controlled from consoles by the main operating surgeon and the assistant operator [2]. With the advancement of this technology, robotic-assisted laparoscopy has grown in popularity during the last few decades, and it is now extensively and thoroughly used in surgical procedures, replacing most open surgeries. The advantages of performing robotic-assisted laparoscopy include a smaller incision [3], faster recovery [4,5] and, consequently, shorter postoperative hospital stay [6,7], better aesthetic outcomes [6], less discomfort [8,9], a decreased risk of infection [2], and no oncological drawbacks in cancer patients [10]. Furthermore, the laparoscope’s enlarged vision allows surgeons to observe anatomical structures in detail and accurately dissect, suture, and repair them [11]. 

On the other hand, several drawbacks linked to this methodology have to be considered. The workspace during laparoscopic surgery is narrower [12] than for open surgery and the field of view is more limited [10]; the difficulty in maintaining hemostasis rises [13]; the extra personnel is costly [14,15]; more work is delegated to humans; and the primary operator and the assistant operator may have communication blunders as they are not staring at the same console [2]. 

Dealing with intraoperative bleeding is one of the most difficult aspects of laparoscopic surgery [16] accounting for 23% of all adverse events [17]. Some efforts have been undertaken in recent years to speed up bleeding identification in endoscopic procedures. In particular, most of the methods detected bleeding by utilizing the RGB space parameters [18] or categorizing pixels into “blood” or “non-blood” using color features. These techniques can process and classify information [19,20,21] using a machine learning approach, such as the Support Vector Machine (SVM) [22], that tries to maximize the distance between elements belonging to different classes, or using deep learning [23] which aims at tackling challenging issues by breaking complex concepts down into smaller ones and portraying them as a nested hierarchy of tasks with various levels of abstraction.

Within the deep learning scenario, convolutional neural networks (CNNs) proved to be the most suitable option to automatically extract features and detect adverse events [24], segment bleeding sources and display them to the surgeon [16], classify the images into bleeding and non-bleeding [25], or for real-time bleeding point location, recognition, and tracking [13,26]. CNNs have a unique design that allows them to interact with images while also leveraging their spatial patterns and being quick to train. This efficiency enables us to train deep and multi-layer networks. As a result, these networks achieve exceptional picture categorization and identification outcomes [27]. When compared to other image classification methods, CNNs require very minimal pre-processing. This implies that, in contrast to traditional methods, the network learns to improve the filters (or kernels) through automatic learning. This freedom from past information and human interference in feature extraction is a significant benefit [28]. However, most of the mentioned research has limited use in real-time videos, or cannot be considered end-to-end, since they require time-consuming additional steps to obtain the final result. In other words, end-to-end CNN architecture allows us to retrieve a solution without implementing further steps, such as stabilizers to minimize jitter and smooth camera path, expensive multi-stage temporal CNNs, or Optical Flow input data so that features may be gathered over numerous frames while leveraging temporal information.

CNN approaches have been employed in minimally invasive surgery for software and hardware-based solutions since enhanced computing power and recent breakthroughs in this field enable a standard computer to comprehend the content of an image or video stream in real time. Among the possible neural network approaches, multi-task learning (MTL) is a strategy that may efficiently solve multiple learning tasks in a CNN unified model. MTL more closely matches the learning process of humans than single-task learning since integrating information across domains is a core principle of human intelligence [29]. MTL is an area of machine learning that adopts a training paradigm in which a shared model learns many tasks at the same time [30]. This strategy increases data efficiency, decreases overfitting by exploiting shared features from multiple tasks, and could improve learning speed by leveraging contextual information [31], hence alleviating deep learning’s renowned drawbacks, i.e., high data availability and computation power [32,33]. On the other hand, learning concepts for numerous tasks introduce problems that are not present in single-task learning. Choosing which tasks to study together is difficult since various tasks may have competing requirements. In this situation, improving a model’s performance on one job could damage performance on another with distinct requirements. 

This study proposes an end-to-end encoder–decoder multi-tasking CNN for joint blood accumulation detection and tool segmentation in laparoscopic surgery to maintain the operating room as clean as possible and, consequently, improve the physicians’ visibility. For this purpose, we employed a shared backbone based on the encoder of the U-Net architecture [34], the gold standard for semantic segmentation in medical images. Two separate branches were instead implemented to classify the blood accumulation event and output the segmentation map. Our main contribution is the introduction of an efficient multi-tasking approach for real-time surgical videos, trained with only RGB images and no other additional information, commonly unavailable in real applications.

The paper is organized as follows: Section 2 describes the system’s architecture (Section 2.1), the dataset (Section 2.2), and the training process and metrics (Section 2.3); Section 3 illustrates and discusses the results obtained; finally, Section 4 summarizes and concludes the study.

## 2. Materials and Methods

**Figure 1:**
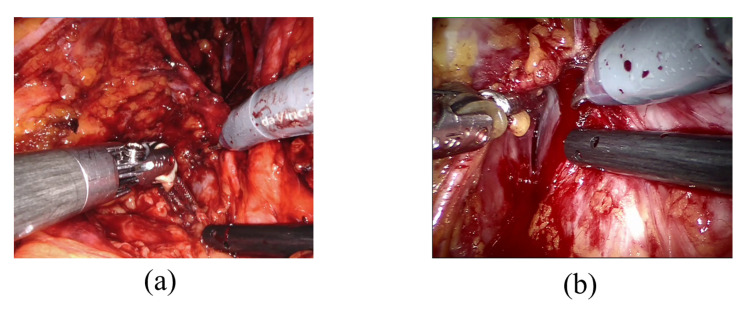
Examples of blood residue on tissues (**a**) and active bleeding (**b**).

The challenge of detecting bleeding in laparoscopic recordings is particularly complex because it requires distinguishing between the presence of blood residual (Figure 1a), which is not an index of bleeding, and when the blood is actively flowing (active bleeding) as shown in Figure 1b, which is the event of interest since it requires surgeon’s prompt intervention. Detecting adverse occurrences during laparoscopic surgeries could be defined as an issue of action detection and localization. Action recognition and object segmentation interactions can be mutually advantageous and increase total video understanding. For example, precise positional identification of the key actor participating in action may boost the robustness of action recognition, and vice versa [31].

The current investigation introduced a multi-task CNN, an architecture able to simultaneously learn multiple tasks. Particularly, in the examined case study, the model could jointly perform semantic segmentation and event detection, namely bleeding identification, in real-time during laparoscopic surgery. To this aim, a new architecture was implemented, fed with a properly manually labeled dataset. A shared trunk architecture was utilized for this purpose, which included a global feature extractor composed of convolutional layers shared by all tasks, followed by a different output branch for each output, performing the same computation for each input of the same task [29]. The weights for multiple tasks were pooled, such that each weight is trained to minimize several loss functions simultaneously. The CNN architecture, the dataset, and the training process are described in the following sections. 

### 2.1. Neural Network Architecture

**Figure 2:**
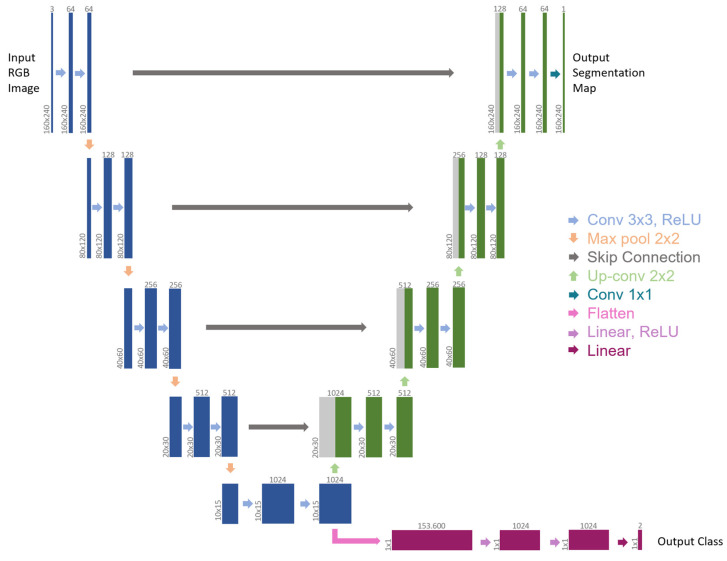
Multi-tasking CNN architecture (example for 160 × 240 input image). Each box represents a multi-channel feature map. The number of channels is provided on top of the box, while the x-y size is shown at the lower left edge. Blue boxes belong to the backbone, green boxes refer to the first branch for semantic segmentation, gray boxes denote copied feature maps (skip connections), and purple boxes represent the second branch for event detection. The arrows refer to the different operations.

Figure 2 illustrates the network architecture. The backbone is a slightly modified version of the U-Net architecture [34]. 

Because of how it is designed, medical data can be analyzed in great detail, making the U-Net a model that is often used in the literature. To deal with RGB images, the number of input channels has been increased in comparison to the original architecture. The contracting path (left side) represents a global feature extraction, and it is shared by all tasks. It comprises a four-time repetition of the same sequence, meaning: two 3 × 3 convolutions (padded convolutions) to double the number of feature channels, each followed by a rectified linear unit (ReLU) and a down-sampling step, made of a 2 × 2 max pooling operation with stride 2, to halve the x-y image size. The last max pooling operation is followed by two 3 × 3 convolutions, which generate the bottleneck of the network. Differently from the original architecture of U-Net, two separate output branches derive from this bottleneck, each addressing a distinct task. The first branch (right-top side) is adapted from the expansive path of U-Net. Here, every step is symmetrical to the related contracting part. It includes an upsampling of the feature map, a 2 × 2 convolution (“up-convolution”) to halve the number of feature channels, a concatenation with the symmetrical feature map of the contracting path (skip connection), and two 3 × 3 convolutions that double the x-y image size, each followed by a ReLU. The final layer of this branch applies a 1 × 1 convolution to map each 64-component feature vector to the desired number of classes in the output segmentation map. Unlike U-Net, padded convolutions were employed so that the output segmentation map and the input RGB image had the same size. 

The second branch is connected to the encoder output, which is the U-Net architecture’s bottleneck. As a result, it uses the U-Net encoder as the backbone for feature extraction and, from a flattened version of the bottleneck as input, it tackles event detection as a classification problem. This branch is based on a sequence of fully connected layers: two Linear layers with 1024 features, each followed by a ReLU and a Dropout Layer, and a final Linear layer that maps each 1024-component feature vector to the desired number of classes. In this instance, two classes could appear in the output: 0 for “*no blood accumulation*” and 1 for “*blood accumulation*”.

### 2.2. Dataset

The images were acquired from 26 endoscopic videos recording Robotic Assisted Radical Prostatectomy (RARP), a laparoscopic procedure conducted to remove the prostate gland and tissues surrounding it in case of prostate cancer. These data were provided by the “Division of Urology, University of Turin, San Luigi Gonzaga Hospital, Orbassano (Turin), Italy”. The length of the procedures’ videos ranged from a few seconds to 15 min. The recordings were initially edited, omitting sections when the endoscope was not within the abdominal cavity and the operational phases where there was no bleeding. This preliminary skimming produced 104 fragments with a length of less than one minute in 70% of cases or a few minutes in the remaining instances. Then, frames were extracted from these surgical pieces, considering a rate of around one frame per second. Finally, samples that may affect CNN training due to poor resolution, or the absence of surgical equipment were removed. As a result, only high-quality frames were included in the final dataset, which comprised 318 images. The dataset was then divided into train, validation, and test sets which contained 200, 32, and 86 images, respectively. Among them, four videos were kept apart for subsequently testing the network in real time.

The training and validation samples were labeled under the supervision of specialized medical personnel, and the results were assessed in the same manner.

**Figure 3:**
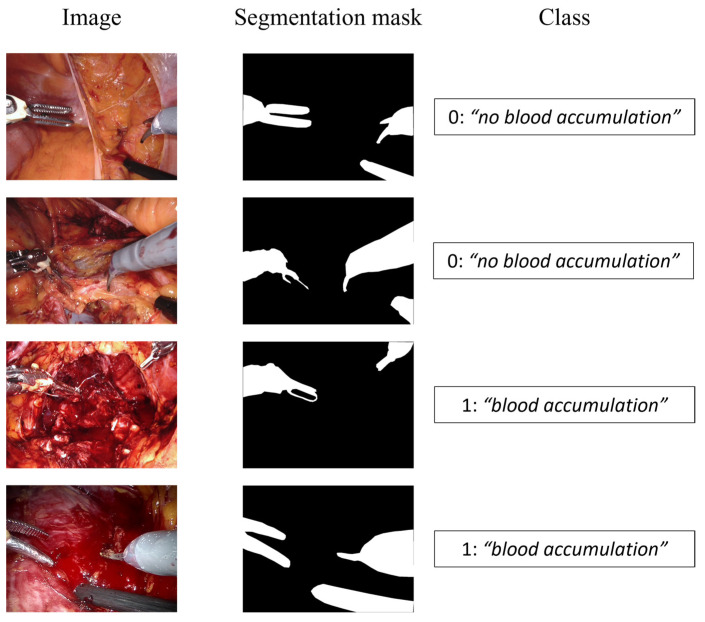
Dataset samples. The first column displays the input image, the second shows the segmentation mask for the semantic segmentation branch, and the third represents the class for the event detection branch.

The images were tagged using two different labels according to the specific task, namely, semantic segmentation and event detection, as shown in Figure 3. 

The semantic segmentation consists in identifying the pixels of the image belonging to the surgical tool, labeling the region of interest (ROI) as “tool” and the other pixels as “background”. On the other hand, the event detection task aims at classifying if the blood is accumulating and an intervention by the surgeon is required, or if the surgical field of view is not affected by a too copious accumulation of blood. To this aim, two possible classes were considered, 0 for “*no blood accumulation*” and 1 for “*blood accumulation*”.

Data augmentation was added during the network training, to improve the numerosity and transformation invariance of the medical image dataset. Particularly, train samples were rotated by a random factor in the range (−35, 35), and randomly flipped, including vertically, horizontally, or both flips.

### 2.3. Training and Metrics

The multi-tasking architecture was trained for 30 epochs using a batch size of 32, and an Adam optimizer with a learning rate of 0.0001. A multi-task loss was chosen for parameter optimization:

(1) $$loss=seg_{loss}+cls_{loss}$$

where $seg_{loss}$ is a Binary Cross Entropy Loss function followed by a *Sigmoid* activation function, and $cls_{loss}$ is a Cross Entropy Loss function followed by a *Softmax* activation function. The model ran on an NVIDIA Quadro P4000 GPU, adopting the open-source *PyTorch* machine learning framework, written in Python, and based on the Torch library.

The semantic segmentation branch accuracy was assessed by the Dice Coefficient (F1 Score) metric, a diffuse metric for semantic segmentation, defined as:

(2) $$Dice\;Coefficient=\frac{2\times Overlap\;Area}{Total\;pixels\;combined}$$

where the overlap area represents the intersection between the pixels belonging to the predicted segmentation masks and those belonging to the ground truth one, and the total pixels parameter represents the total number of pixels in both images. The Dice Coefficient ranges from 0 to 1, where the edge values mean completely wrong and perfectly correct predictions, respectively.

The event detection branch accuracy was calculated as follows:

(3) $$Classification\;Accuracy=\frac{\#correct\_predictions}{\#samples}$$

This value was monitored both to examine the accuracy of the entire branch, and the accuracy of each class separately.

## 3. Results and Discussion

**Figure 4:**
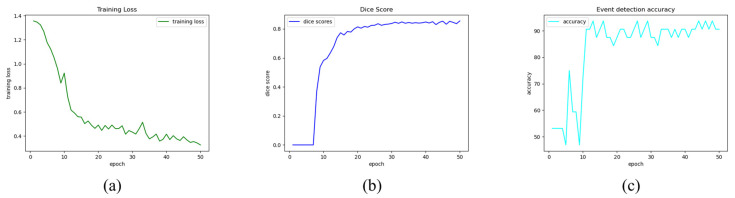
Training and validation metrics trends. Multi-task Training Loss (**a**), Validation Dice Score for semantic segmentation branch (**b**), and Validation accuracy for event detection branch (**c**).

The implemented multi-task CNN was tested both on images and videos. For each epoch the training loss (Figure 4a) and the validation metrics, namely Dice Score (Figure 4b) and event detection accuracy (Figure 4c), were plotted to show their trend. Following that, epoch 30 was picked for the final model since it was deemed the optimal tradeoff between the two branches of the network according to the experimental tests on images and videos.

Concerning the tests on images, the multi-task CNN achieved a Dice Score equal to 81.89% for the semantic segmentation task, and an accuracy equal to 90.63% for the event detection task without any pre- or postprocessing step. Furthermore, the accuracy for each class was detailed, obtaining an accuracy of 86.67% and 94.12% for classes “*no blood accumulation*” and “*blood accumulation*”, respectively. 

Afterward, the network was also tested on videos to assess its real-time performance. Starting from test videos with a resolution of 1280 × 720 and a frame rate of 30 frames-per-second, the model output a processed video stream with 15 frames-per-second, when the prediction was performed for each frame. Comparative real-time and accuracy tests were carried on, and the network output was experimentally observed at different prediction frequencies, namely, the number of frames between one prediction and the next one. A test input video stream with a frame rate of 30 frames per second was employed to accomplish this test. For the real-time comparison tests, ten distinct values were investigated, as given in Table 1, with each indicating the prediction frequency. 

**Figure 5:**
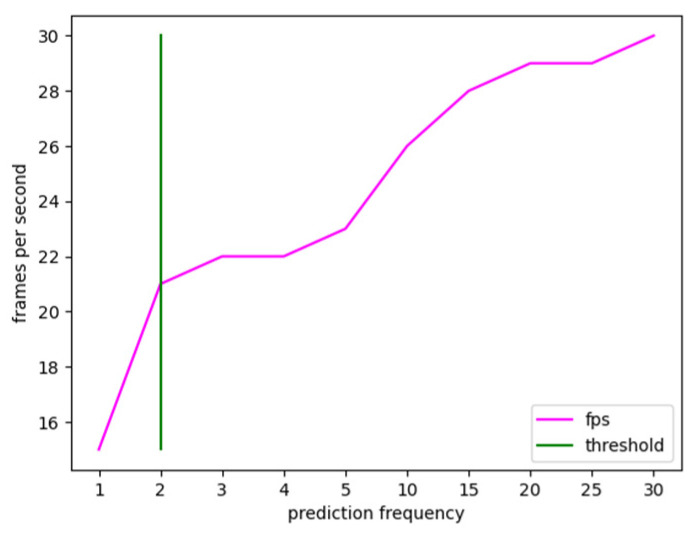
Frame rate trend versus prediction refresh rate calculated on a test video stream. The value 2 was experimentally chosen as the optimal tradeoff between real-time and accuracy.

The condition under which the prediction is made on all frames of the video stream was chosen as the minimal value, which is one frame. Instead, 30 frames were determined as the largest possible value, assuming one prediction each second. Higher values were not examined because, as previously stated, active bleeding necessitates immediate action; hence, an update rate of one second was deemed a limiting number. As seen in the table, just one prediction per second is required for the processed video stream to have the same frame rate as the input stream. However, it has been observed experimentally that the accuracy reduces considerably, particularly for segmentation masks, due to the rapid movement of the surgical instruments. In contrast, when the prediction frequency is lowered, there is a wider tolerance in terms of the percentage of blood accumulation predicted. The experimental findings show that lowering the forecasts by 50% and making a prediction every two frames delivers an increase in frame rate without impacting prediction accuracy; hence, it was regarded as the ideal threshold, as shown in Figure 5. 

As a result, the 21-frames-per-second limitation was assessed by the medical equipment to be the upper limit as the trade-off between real-time and accuracy. In other words, further increasing the frame rate at the expense of accuracy was considered unacceptable. 

**Figure 6:**
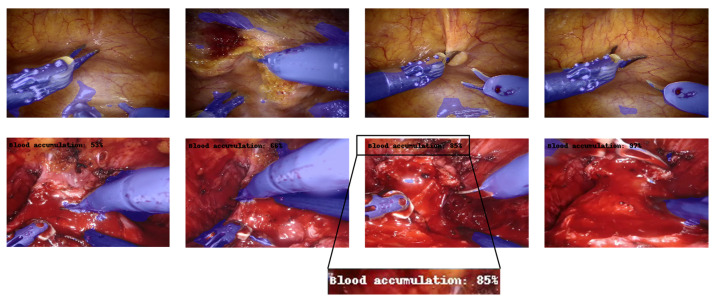
Multi-tasking CNN architecture (example for 160 × 240 input image). Each box represents a multi-channel feature map. Tools detected are blue-colored, while the blood accumulation percentage is indicated in the top-left corner of the image. The first row contains samples for which the percentage of blood accumulation is not displayed because the model predicted a value of less than 50%. The second row, in contrast, displays samples with a blood accumulation percentage greater than 50%.

Figure 6 shows some examples of output frames, containing the output segmentation map overlapped on the real counterpart, and the predicted percentage of the “*blood accumulation*” class, if it is greater than 50%. As can be seen from the figure, both branches provided satisfactory results. The CNN succeeded, although with some marginal imperfections, in correctly recognizing the surgical tools and overlaying the mask. In addition, the event related to blood accumulation was also properly detected, as the percentage goes above 50% when bleeding is effectively visible. 

**Figure 7:**
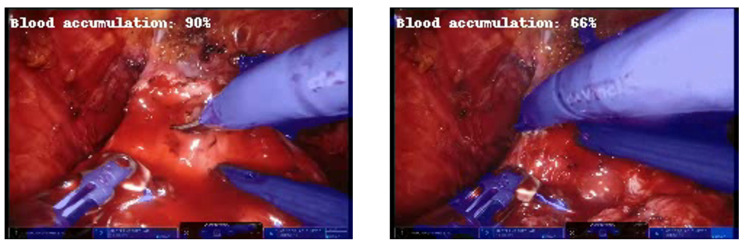
Example of frames at about 3 s distance in which the predicted percentage of blood accumulation decreases as the aspirator removes excess blood.

Furthermore, it is noteworthy that during the test on the videos, the network adequately recognized the reduction in accumulated blood in the operating scene as the percentage decreased when the laparoscopic aspirator was removing the accumulated blood (Figure 7).

It can be inferred that tasks to be solved simultaneously were chosen properly since the features extracted from the shared backbone for tool segmentation also proved worthwhile for event detection since the two tasks are related. In fact, in most cases, active bleeding occurs at the time when one of the tools closes or interacts with anatomical structures such as the prostate. On the other hand, there is a reduction in accumulated bleeding when the aspirator starts to extract the blood from the surgical field. However, it was noted that this mutual benefit was lost when the number of epochs increased. In this situation, there was an improvement in accuracy relative to semantic segmentation, but the event detection task degenerated. This situation according to which the accuracy related to one task increases at the expense of the accuracy-related to the other task is not uncommon and, as already mentioned, is a known issue in the literature.

It was also possible to make assumptions about the reasons for the network’s flaws because frames taken from recordings of actual interventions were investigated. Particularly when the light is changing, it could be challenging to distinguish between surgical instruments. In those circumstances, active bleeding might be misinterpreted with passive bleeding on the walls or on other anatomical parts within the surgical field. 

The achieved results fulfilled the aim of using the same CNN architecture to simultaneously identify surgical tools in the field of view and detect the bleeding in real time. Future work is going to be planned to proceed in four different directions:

1. Neural Network Architecture. The architecture of the network should be extended to consider temporal information extracted from sequential images. This improvement will likely enhance the branch related to event detection in terms of accuracy and reliability. In this research context, for example, it may be easier to distinguish passive bleeding, namely, blood residue on tissues, and active bleeding, which is the surgeons’ object of interest. Moreover, the semantic segmentation task should distinguish tools with different labels to improve the prediction, while the detection task should be extended by adding the localization of the origin of bleeding, which may provide remarkable clinical advantages when the human eye cannot instantly catch it [35].
2. Dataset. An improved dataset in terms of numerosity and variance could be beneficial to increase the accuracy of prediction. Furthermore, the sequences of images that do not contain any structure of interest (for instance, the external view of the operating room, and the images inside the trocar) in a limited-size dataset might improve the knowledge of the network about the studied environment [35]. Alternatively, from an algorithmic perspective, it could be advantageous to provide depth information as well as RGB information, to improve the tools’ tracking accuracy. To accomplish this advancement, 3D acquisition cameras should be integrated with the RGB cameras employed during surgical interventions.
3. Testing. The model should be evaluated in the operating room to determine its practical limitations in the setting of a real-time application and then enhanced accordingly by adding new features.
4. Research field. Extending the algorithm into different domains would be of interest, to perform further analysis both in terms of actor segmentation and tracking and from the point of view of event detection and classification.

## 4. Conclusions

This study implemented an end-to-end encoder-decoder multi-tasking CNN for joint blood accumulation detection and tool segmentation in laparoscopic surgery. One of the most problematic aspects of laparoscopic surgery is dealing with intraoperative bleeding. Since the operation area is constantly limited and blood rapidly fills the bleeding site, it is difficult to control bleeding during laparoscopic procedures, indeed any sort of surgical manipulation, including suction, grabbing, retraction, cutting, and dissection might result in rapid bleeding and immediate treatment is required to avoid significant consequences.

To the best of our knowledge, there are no other systems that address simultaneously both the surgical tools identification and the event detection, namely the bleeding detection, in an end-to-end fashion and with only RGB images as input. The current study’s results suggested that multi-task learning may be a remarkable strategy for improving efficiency and performance by employing shared features from multiple tasks. In this sense, maintaining a high level of accuracy and preserving the real-time is of utmost importance to make the methodology suitable to be used in the surgical room and support surgeons during the interventions.

The obtained findings allow us to deal with real-time data performing more tasks at the same time and achieving a noteworthy trade-off between accuracy and performance. Future research is indeed aimed at enhancing the system in the following aspects: temporal information of sequential pictures could be taken into consideration to improve the accuracy (a new architecture adaptation will be needed), the dataset could be expanded to increase the variability of the data and improve the neural network generalization, and the test should be performed on live surgeries to tune the CNN parameters, although considerable changes are not expected since the current work has already used data provided by real interventions. Moreover, it would be desirable to involve other domains to produce a generalizable real-time framework helpful for applications in several disciplines.

## Figures and Tables

**Table 1:** Comparison between prediction rate and frame rate during video tests.

Prediction Frequency	Frames-per-Second
1	15
2	21
3	22
4	22
5	23
10	26
15	28
20	29
25	29
30	30

## Data Availability

No new data were created in this study. Data sharing is not applicable to this article.
